# Echinatin mitigates sevoflurane-induced hippocampal neurotoxicity and cognitive deficits through mitigation of iron overload and oxidative stress

**DOI:** 10.1080/13880209.2022.2123941

**Published:** 2022-10-07

**Authors:** Zilong Xu, Yanqiu You, Qiuqin Tang, Hui Zeng, Tianshou Zhao, Juan Wang, Fujun Li

**Affiliations:** a Department of Anesthesiology, The First Affiliated Hospital of Harbin Medical University, Harbin, China; b Department of Laboratory Medicine, The Ruikang Hospital Affiliated to Guangxi University of Chinese Medicine, Nanning, China; c Department of Anesthesiology, The Ruikang Hospital Affiliated to Guangxi University of Chinese Medicine, Nanning, China

**Keywords:** Anaesthesia, postoperative cognitive dysfunction, hippocampal neuron, ferroptosis

## Abstract

**Context:**

Sevoflurane (Sev) is a commonly used surgical anaesthetic; it has neurotoxic effects on the brain. Echinatin (Ech) is reported to have anti-inflammatory and antioxidant activity.

**Objective:**

This research confirms the effect of Ech on Sev-induced neurotoxicity and cognitive deficits.

**Materials and methods:**

Primary rat hippocampal neurons were treated with 4.1% Sev for 6 h in the presence of Ech (5, 10, and 20 μM) or vehicle, followed by a further 42 h of culture. Male Sprague-Dawley aged rats were divided into 6 groups (*n* = 6): control, Sev, Sev + Ech (20 mg/kg;), Sev + Ech (40 mg/kg), and Sev + Ech (80 mg/kg). Rats were intraperitoneally injected with Ech or vehicle 1 h before Sev exposure (2% Sev for 5 h).

**Results:**

We found that Ech (5, 10, and 20 μM) elevated cell viability (1.29-, 1.51-, 1.68-fold) but mitigated apoptosis (23.87% vs. 16.48%, 12.72%, 9.02%), oxidative stress, and ferroptosis in hippocampal neurons with Sev treatment. Ech activated the Nrf2 expression in Sev-induced *in vitro* and *in vivo* models of anaesthetic neurotoxicity. Ech also weakened neurotoxicity in hippocampal neurons with Sev treatment by increasing Nrf2 expression level. Moreover, Ech alleviated hippocampus neurons apoptosis (19.38% vs. 16.05%, 11.71%, 8.88%), oxidative stress, and ferroptosis in rats with Sev treatment. Ech improved Sev-induced cognitive deficits in rats.

**Conclusions:**

Ech alleviates Sev-induced neurotoxicity and cognitive deficits by mitigation of ferroptosis and oxidative stress. Ech may be developed as a new promising therapeutic drug for treatment of cerebral nerve injury caused by surgical anaesthesia.

## Introduction

Sevoflurane (Sev) is a widely used inhalation anaesthetic in surgery. Sev is characterised by rapid onset of anaesthesia and short recovery time (Flick et al. 2011; Xie and Wang 2018). However, Sev has been reported to produce toxic effects on cerebral neurons (Zhou et al. 2020). To make matters worse, the neurotoxicity of anaesthesia can affect the production of fast synapses, and this effect can last for years (Xie and Wang 2018). Many critically ill elderly sufferers sometimes need repeated surgical interventions, so it is vital to study the effects of repeated exposure to anaesthetic (such as Sev) on learning and memory. It has been reported that repeated exposure to Sev impairs learning and memory in aged male rats, and this impairment is accompanied by cognitive related biochemical changes in the hippocampus (Guo et al. 2018). Therefore, it is urgent to find more effective drugs and understand their mechanisms to reduce the neurotoxicity and cognitive impairment caused by Sev.

Echinatin (Ech) is a liquorice flavonoid which has anti-inflammatory and antioxidant activity (Honda et al. 2014). Previous research has found that Ech displayed a protective effect against ischemia/reperfusion-induced damage of myocardial cells (Tian et al. 2016). In addition, it was demonstrated that Ech reduced the levels of reactive oxygen species (ROS), interleukin-6, and prostaglandin E2 in LPS-stimulated macrophage cells, thus exerting its antioxidant and anti-inflammatory effects (Fu et al. 2013). Ech efficiently suppressed the activation of NLRP3 inflammasome to exert a protective effect against NLRP3-driven sicknesses (Xu et al. 2021). Moreover, Ech alleviated H_2_O_2_-induced apoptosis and oxidative injury in human lens epithelial B3 cells (HLECs) by modulating the Nrf2/HO-1 signalling (Ran et al. 2021). Consequently, we speculated that Ech may attenuate the neurotoxicity induced by Sev anaesthesia via the anti-apoptotic and antioxidant pathways. In this research, we carried out *in vivo* and *in vitro* experiments to investigate the influence of Ech on Sev-induced neurotoxicity in hippocampal neurons and explore the underlying molecular mechanisms.

This research uncovered the functions of Ech in neurotoxicity and cognitive deficits induced by Sev anaesthesia, and further shed light on the neuroprotective mechanism of Ech in primary rat hippocampal neurons and the Sev-treatment rat model.

## Materials and methods

### Cell lines and treatment

Primary rat hippocampal neurons were obtained from Procell Life Science & Technology Co., Ltd. (Wuhan, China). These cells were cultured in Dulbecco’s modified Eagle’s medium/F12 (51445 C; Sigma-Aldrich, St. Louis, MO, USA) with 2% B-27 and 10% FBS (12103 C; Sigma-Aldrich) in 5% CO_2_ at 37 °C. To simulate the state of cells under anaesthesia, hippocampal neurons were treated with 4.1% Sev (Y0001046; Sigma-Aldrich) for 6 h with vehicle or Ech (5, 10, or 20 μM; PHL83847; Sigma), followed by a further 42 h of culture in a 5% CO_2_ incubator. Ech was dissolved in DMSO (D2650; Sigma-Aldrich) and then diluted with phosphate buffer saline. For the control and Sev groups, an equal volume of vehicle was added to the hippocampal neurons.

### Cell viability assay

After Sev treatment, hippocampal neurons were cultured for 42 h in 96-well plates in the presence or absence of Ech (5, 10, or 20 μM). These cells were exposed to 5 mg/mL 3-(4, 5-dimethylthiazol-2-yl)-2, 5-diphenyltetrazolium bromide (MTT) solution (20 μL/well; 11465007001; Sigma-Aldrich) for 4 h, followed by incubation of solubilisation solution. Finally, the absorbance value of each well at 490 nm was examined with a microplate reader (Thermo Fisher Scientific, Waltham, MA, USA).

### Determination of lactate dehydrogenase (LDH), malondialdehyde (MDA), and glutathione (GSH) levels

The hippocampal neurons and tissues were lysed and the lysate was centrifuged for determining the levels of LDH (ab102526; Abcam, Cambridge, MA, USA), MDA (ab118970; Abcam), and GSH (ab112132; Abcam) by matching commercial kits. All operation steps refer to the instructions of these kits.

### Terminal deoxynucleotidyl transferase (TdT) dUTP Nick-End labeling (Tunel) assay

To assess cell apoptosis, hippocampal neurons were planted into 6-well plates. Afterwards, a TUNEL in Situ Cell Death Detection Kit (11684817910; Sigma-Aldrich) was used to assess cell apoptosis. Hippocampal neurons were incubated with TUNEL (5 µM; Sigma-Aldrich) and these cell nuclei were stained with 4′,6-diamidino-2-phenylin-dole (D9542; Sigma-Aldrich). In the last, a fluorescent microscope (Leica, Shanghai, China) was utilised to observe these cells. For hippocampal tissues of rats, five tissue slices of each specimen were taken for apoptosis detection. For each slice, a single region of interest within hippocampal CA1 region was randomly selected to take photos. All counts in the Tunel assay were done by an independent investigator in a blinded manner.

### Measurement of ROS level

ROS content was evaluated by utilising a DCFH-DA fluorescent probe (MAK143; Sigma-Aldrich). The H_2_O_2_ in cells can oxidise DCFH-DA to a highly fluorescent compound, dichlorofluorescein (DCF). After diverse treatments, hippocampal neurons were exposed to 10 mM DCFH-DA (Sigma-Aldrich) for 30 min. After washing, the fluorescence of the hippocampal neurons was assessed by a microplate reader (Thermo Fisher Scientific) or a fluorescence microscope (Leica).

### Iron assay

In order to detect the iron level in hippocampal neurons, an iron assay kit (ab83366; Abcam) was applied in line with the instructions.

### Western blot

Tissues or cells were lysed and Pierce BCA Protein Assay Kit (71285-M; Sigma-Aldrich) was used for protein quantification. In some experiments, nuclear protein extraction was conducted by a nuclear extraction kit (ab219177, Abcam). Then, total protein (50 μg) was separated by 12% sodium dodecyl sulphate polyacrylamide gel electrophoresis and moved to polyvinylidene difluoride membranes (IPVH85R; Sigma-Aldrich). Then, the membrane was blocked and incubated with primary antibodies overnight at 4 °C. The antibodies were as follows: anti-ferritin (ab75973; 1:1,000; Abcam), anti-GPX4 (ab125066; 1:1,000; Abcam), anti-Nrf2 (AF0639; 1:1,000; Affinity Biosciences, Changzhou, China), anti-β-actin (AF7018; 1:1000; Affinity), and anti-Lamin B1 (BF8009; 1:1,000; Affinity). The membrane was incubated with the goat anti-rabbit IgG (ab205718; 1:2500; Abcam) for 1 h at 25 °C. The protein bands were detected by a chemiluminescence kit (11520709001; Sigma-Aldrich) and analysed by ImageJ software (NIH, Bethesda, MD, USA).

### Real-time quantitative PCR (qRT-PCR)

Trizol (93289; Sigma-Aldrich) was used to extract RNA. The total RNA was applied for the synthesis of cDNA by reverse transcription reaction. Fast Start Universal SYBR Green Mastermix (FSUSGMMRO; Sigma-Aldrich) was employed to perform qRT-PCR. β-actin served as the housekeeping gene. The 2^−ΔΔCt^ method was employed to estimate mRNAs abundances. All primer sequences were as follows: Nrf2 forward and reverse primers were 5′-TGACTCTGACTCCGGCATTTCACT-3′ and 5′-TCCATTTCCGAGTCACTGAACCCA-3′. β-actin forward and reverse primers were 5′-CCCGCGAGTACAACCTTCTT-3′ and 5′-CGCAGCGATATCGTCATCCA-3′.

### Cell transfection

The small interfering RNA (siRNA) binding Nrf2 (si-Nrf2) and control (si-NC) were purchased from GenePharma (Shanghai, China). Lipofectamine 3000 (Invitrogen, Carlsbad, CA, USA) was applied to perform cell transfection in keeping with the operating instructions.

### Animal test

The Sprague-Dawley rats (male, 20-month-old, 550–700 g*, n* = 6 per group) were obtained from the Beijing Vital River Laboratory Animal Technology Co., Ltd. (Beijng, China). A total of 78 rats were used in animal experiments. Rats were allocated into the following five experimental groups: control, Sev, Sev + Ech (L), Sev + Ech (M), and Sev + Ech (H). Ech (Sigma-Aldrich; purity ≥ 98%) was given to rats by intraperitoneal injection as a single dose of 20 (L), 40 (M), or 80 mg/kg (H) at 1 h before Sev exposure. The injection volume of each rat was 5 mL. For control and Sev groups, an equal volume of vehicle was intraperitoneally injected into rats. Then, rats except for the control group were anaesthetised with 2% Sev (Sigma-Aldrich) for 5 h. The histological and biochemical analysis of the hippocampus was done 48 h later after the rats were sacrificed and the brains were removed. The separation of hippocampus of rats was performed according to a previous paper (Yilmaz et al. 2021). The *in vivo* tests were allowed by animal ethics committee of The First Affiliated Hospital of Harbin Medical University (No. SYXK2021004).

### Hematoxylin-Eosin (HE) staining

The brains of rats were immersed in formalin solution (4%; HT501128; Sigma-Aldrich) for 8 h at 4 °C. Whereafter, these tissues were soaked with ethanol solution (70%; E7023; Sigma-Aldrich) for 5 min, and then soaked in 80%, 90%, 95%, and absolute ethanol (Sigma-Aldrich) to accomplish gradient dehydration. Lastly, the above tissues were permeated with xylene (214736; Sigma-Aldrich) for 0.5 h, and then embedded in paraffin (327204; Sigma-Aldrich). The rat brains were cut into slices with a thickness of 3 μm. Five tissue slices of each specimen were taken for HE detection. For each slice, a single region of interest within hippocampal CA1 region was randomly selected to take photos.

### Cognitive function assay

In this study, the cognitive ability of rats was assessed by Morris Water Maze (MWM) assay. MWM experiment is a commonly used tool for studying spatial learning and memory abilities in rats and mice, especially the hippocampal-dependent cognitive ability (Lv et al. 2020). The experimenters were not informed of the grouping of the rats. Rats were allowed to recover for 48 h after Sev anaesthesia. Subsequently, rats in the control group and Sev group were trained for 5 uninterrupted days. The test site was a circular pool consisting of four quadrants. In one of the quadrants, we placed an invisible transparent escape platform. The rats got into the field randomly from one of the quadrants and were permissible to discovery the invisible platform. Four trials were implemented at different quadrant entrances on each training day. The escape latency was recorded using a video tracking system. After the training, we removed the transparent platform. Video tracking system was used to observe the time spent in the target quadrant, crossing platform times within 60 s, and swim speed of rats.

### Statistical assay

All statistical analysis was performed by SPSS 22.0, based on no less than three repetition for every test. All figures were presented as means ± standard deviation (*SD*). One-Way or Two-Way Analysis of Variance (ANOVA) followed by Turkey’s *post hoc* test was the way used for the examination of significant difference. It was considered statistically significant when *p* < 0.05.

## Results

### Ech mitigates Sev-induced apoptosis of hippocampal neurons

**Figure 1.**
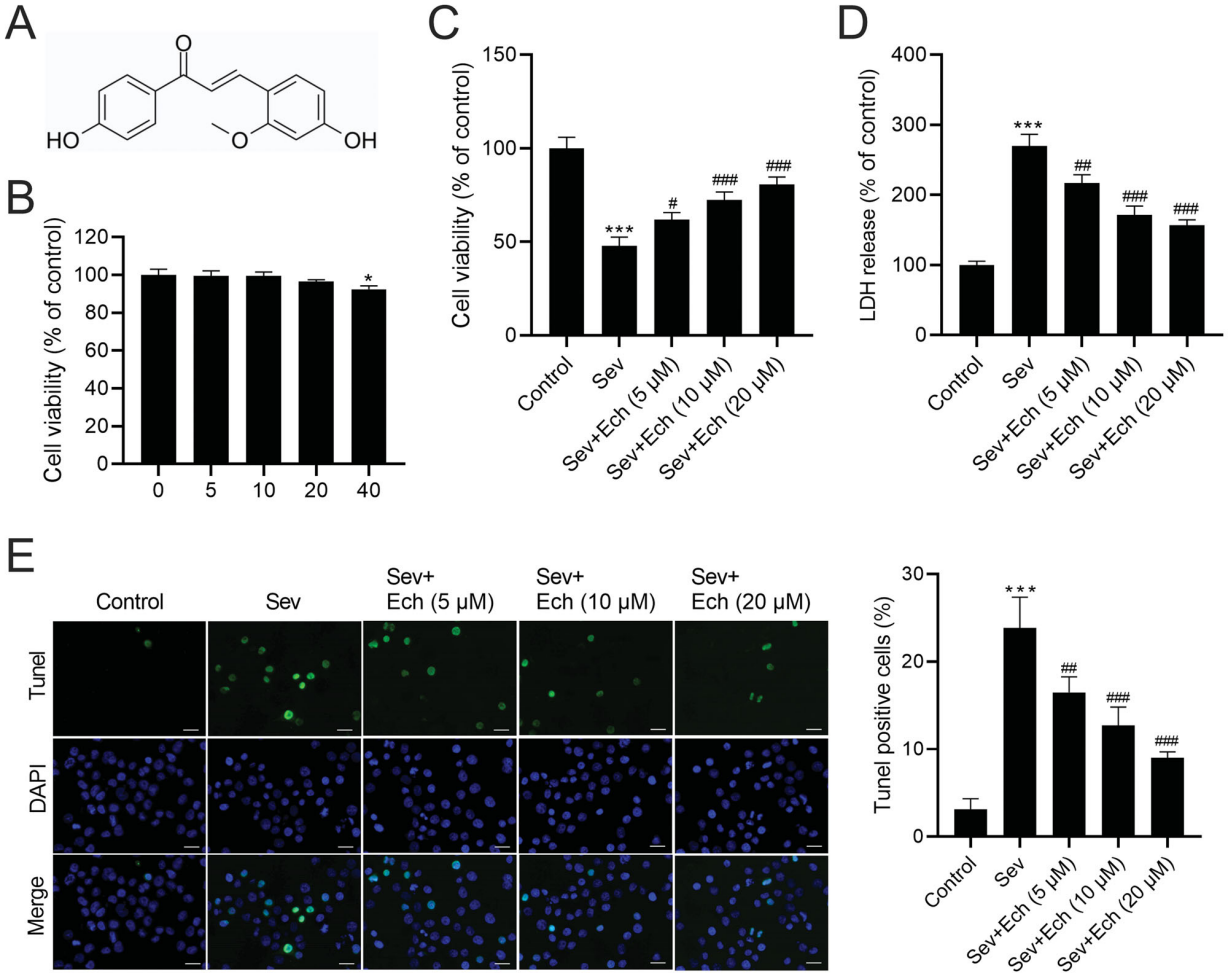
Ech mitigates Sev-induced apoptosis in hippocampal neurons. (A) The chemical structure of Ech. (B and C) The cell vitality was analysed by MTT assay. (D) The LDH release was detected by the commercial kit. (E) The apoptosis was assessed by the Tunel assay. Compared with the control group, *p < 0.05, ***p < 0.001. Compared with the Sev group, ^#^*p* < 0.05, ^##^*p* < 0.01, ^###^*p* < 0.001.

In this part, we assessed the effects of Ech on hippocampal neurons with Sev treatment. The chemical structure of Ech was shown in Figure 1(A). To confirm the cytotoxicity of Ech, the hippocampal neurons were exposed to diverse concentrations of Ech (0, 5, 10, 20, or 40 μM) for 48 h. Then, we found that viability of hippocampal neurons was decreased after 40 μM Ech treatment (Figure 1(B)). Consequently, the 5, 10, and 20 μM Ech were used in the next tests. The hippocampal neurons were then divided into 5 groups. For the control and Sev groups, an equal volume of vehicle was added to the hippocampal neurons. In the Sev group, hippocampal neurons were treated with 4.1% Sev for 6 h, followed by a further 42 h in a 5% CO_2_ incubator. In the Sev + Ech groups, after the addition of diverse concentrations of Ech (5, 10, or 20 μM), hippocampal neurons were immediately treated with 4.1% Sev for 6 h, followed by a further 42 h in a 5% CO_2_ incubator. We found that viability of hippocampal neurons was decreased by Sev treatment, which was weakened by Ech treatment (Figure 1(C)). Moreover, the levels of LDH release (Figure 1(D)) and apoptosis rate (Figure 1(E)) were obviously increased by Sev treatment, while these results were attenuated by Ech treatment. This evidence indicated that Ech elevated cell viability but reduced cell apoptosis in hippocampal neurons with Sev treatment.

### Ech restrains Sev-induced oxidative stress and ferroptosis in hippocampal neurons

**Figure 2.**
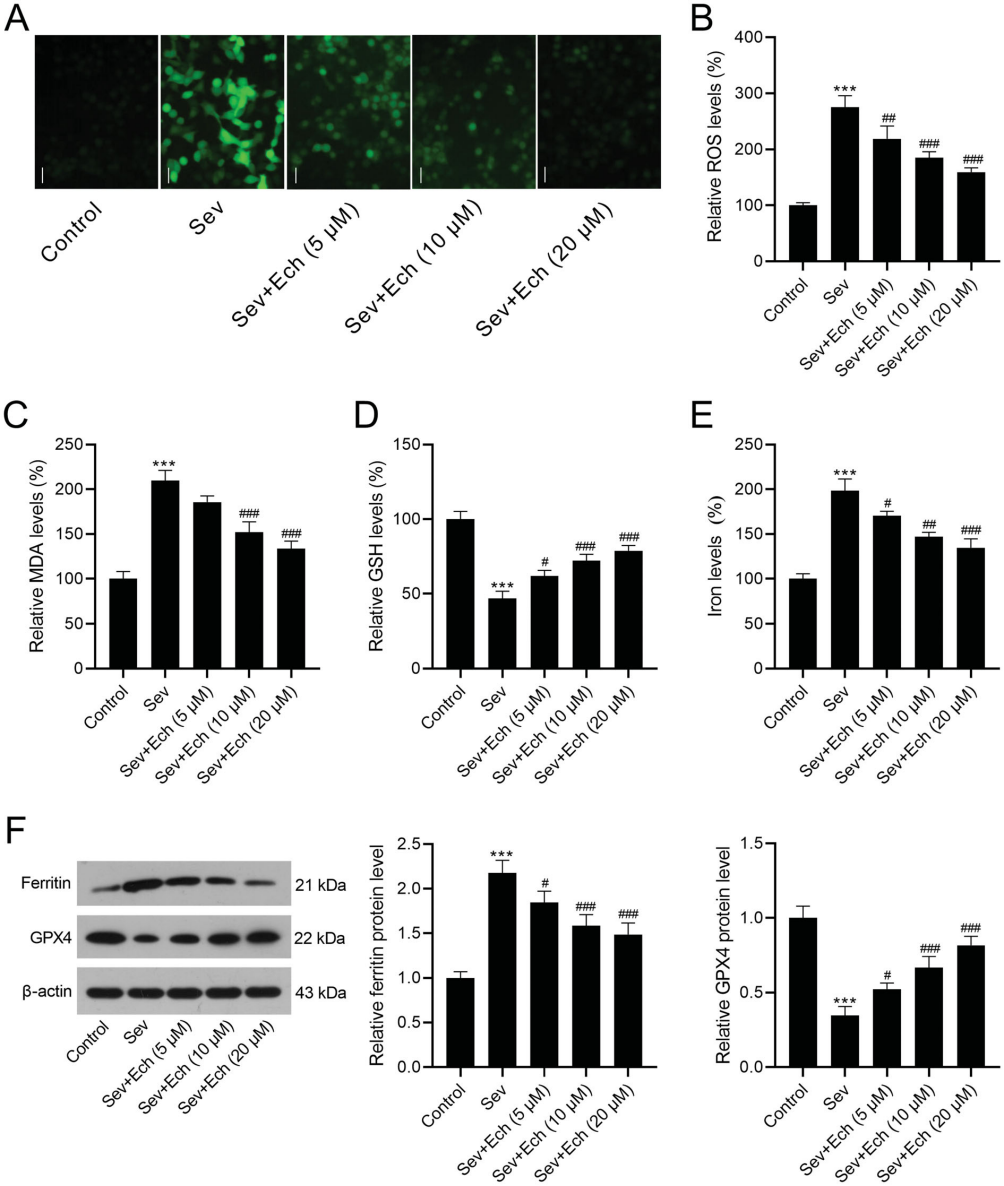
Echinatin regulates Sev-induced oxidative stress and ferroptosis in hippocampal neurons. (A and B) The level of ROS was evaluated by a commercial kit. (C–E) The levels of MDA, GSH, and iron were detected by commercial kits. (F) The ferritin and GPX4 levels were detected by western blot. Compared with the control group, ***p < 0.001. Compared with the Sev group, ^#^*p* < 0.05, ^##^*p* < 0.01, ^###^*p* < 0.001.

Then, we investigated the effects of Ech on oxidative stress and ferroptosis of hippocampal neurons. Results showed that the levels of ROS (Figure 2(A,B)) and MDA (Figure 2(C)) in hippocampal neurons were increased after Sev treatment, but this consequence was weakened after Ech treatment. On the contrary, the GSH level was decreased after Sev treatment, whereas this influence was attenuated by Ech treatment in hippocampal neurons (Figure 2(D)). On the other hand, the iron level in hippocampal neurons was increased by Sev treatment, however this elevation was attenuated after Ech treatment (Figure 2(E)). Subsequently, western blot was performed to detect the levels of related proteins. The ferritin level was increased but GPX4 level was decreased in hippocampal neurons with Sev treatment, but these changes were weakened after Ech treatment (Figure 2(F)). Therefore, these findings indicated that Ech inhibited oxidative stress and ferroptosis in hippocampal neurons with Sev treatment.

### Ech activates Nrf2 in hippocampal neurons and hippocampus of aged rats

**Figure 3.**
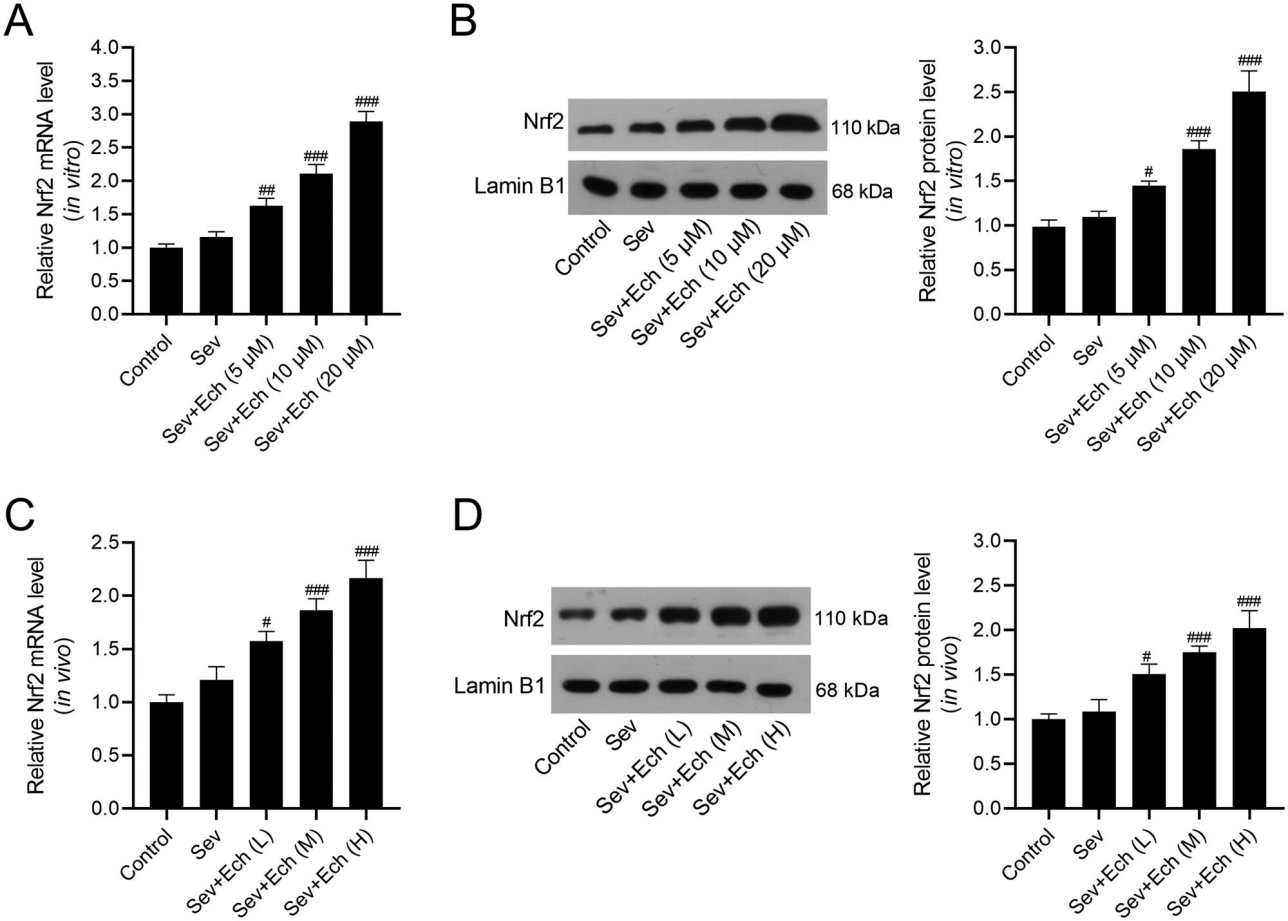
Ech activates Nrf2 in hippocampal neurons and hippocampus of aged rats. (A and B) The mRNA and protein levels of Nrf2 *in vitro* were detected by qRT-PCR and western blot. (C and D) The mRNA and protein levels of Nrf2 *in vivo* were detected by qRT-PCR and western blot. Compared with the Sev group, ^#^*p* < 0.05, ^##^*p* < 0.01, ^###^*p* < 0.001.

Next, we explored whether the protective activity of Ech against Sev-induced neurotoxicity by modulating Nrf2. Results showed that the level of Nrf2 mRNA in hippocampal neurons (Figure 3(A) and Nrf2 protein levels in cell nuclei (Figure 3(B)) were slightly elevated by Sev treatment, but no statistical significance was observed. The Nrf2 mRNA and protein levels were increased by Ech treatment *in vitro* (Figure 3(A,B)). In the *in vivo* experiments, the levels of Nrf2 mRNA in hippocampus of rats (Figure 3(C)) and Nrf2 protein level in hippocampus cell nuclei of rats (Figure 3(D)) were also slightly elevated by Sev treatment, but no statistical significance was observed. Ech treatment up-regulated the levels of Nrf2 mRNA and protein in hippocampus of rats anaesthetised with Sev. Hence, these results demonstrated that Ech treatment enhanced the Nrf2 expression *in vitro* or *in vivo* under the condition of Sev-induced anaesthetic injury.

### Ech restraines Sev-induced oxidative stress and ferroptosis in hippocampal neurons through the Nrf2 signalling

**Figure 4.**
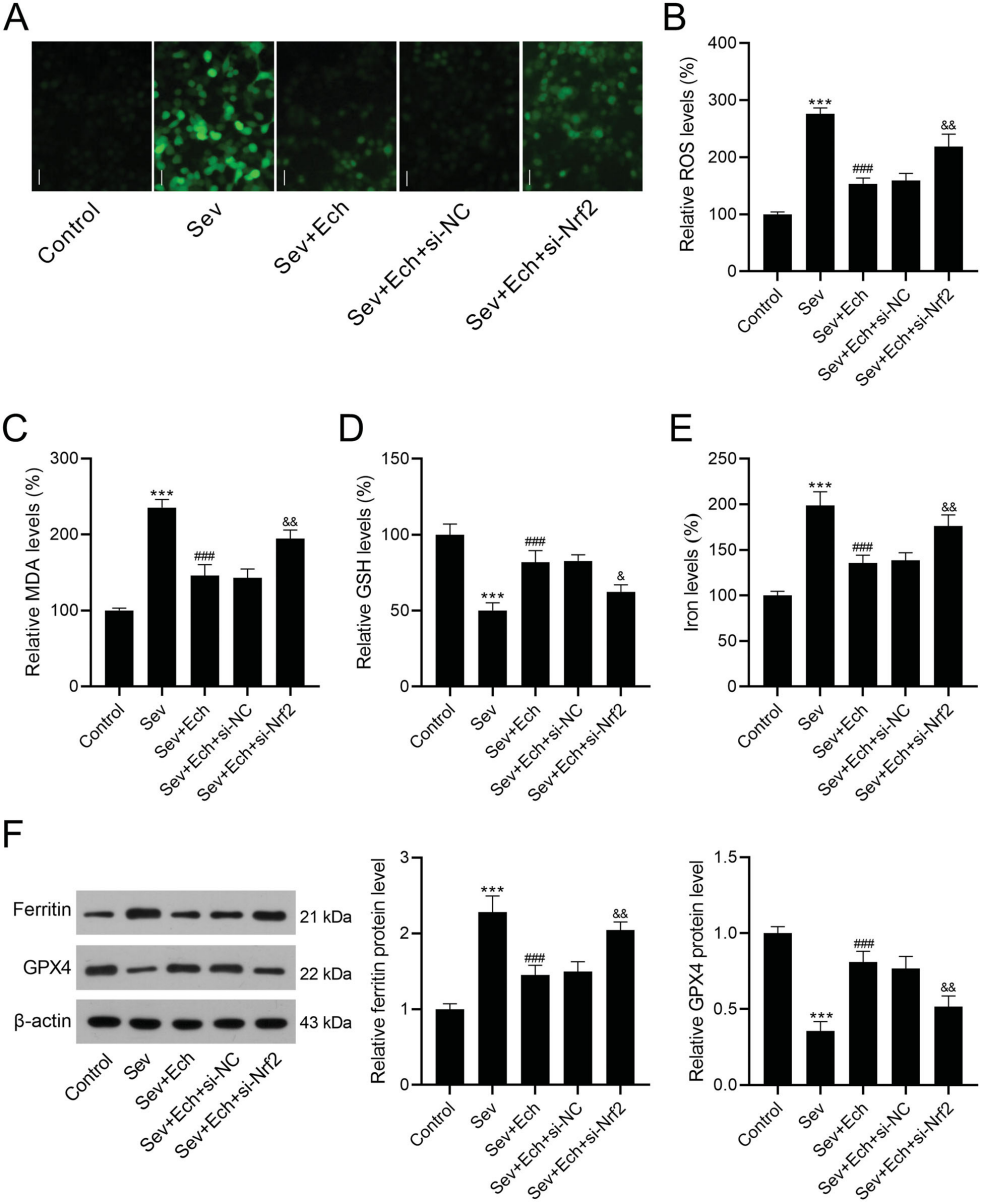
Ech mitigates Sev-induced oxidative stress and ferroptosis in hippocampal neurons via the Nrf2 signalling. (A and B) The level of ROS was detected by a commercial kit. (C-E) The levels of MDA, GSH, and iron were detected by commercial kits. (F) The ferritin and GPX4 protein levels were detected by western blot. Compared with the control group, ***p < 0.001. Compared with the Sev group, ^###^*p* < 0.001. Compared with the Sev + Ech + si-NC group, ^&^*p* < 0.05, ^&&^*p* < 0.01.

Herein, we investigated whether Ech regulated oxidative stress and ferroptosis in hippocampal neurons by the Nrf2 signalling. In the Sev + Ech + si-NC and Sev + Ech + si-Nrf2 groups, hippocampal neurons were transfected with si-NC or si-Nrf2. After the addition of Ech (20 μM), hippocampal neurons were immediately treated with 4.1% Sev for 6 h and then cultured for another 42 h in a 5% CO_2_ incubator. We found that the levels of ROS (Figure 4(A,B)), MDA (Figure 4(C)), and iron (Figure 4(E)) were reduced by Ech treatment, but this effect was weakened by si-Nrf2 transfection in hippocampal neurons with Sev treatment. In contrast, the GSH content was increased after Ech treatment, while this influence was attenuated by silencing Nrf2 in hippocampal neurons with Sev treatment (Figure 4(D)). The level of ferritin was decreased but GPX4 level was increased by Ech treatment, while this impact was attenuated by downregulation of Nrf2 in hippocampal neurons with Sev treatment (Figure 4(F)). In this part, these data supported that Ech restrained oxidative stress and ferroptosis in hippocampal neurons with Sev treatment by modulating the Nrf2 signalling.

### Ech mitigates Sev-induced hippocampal neuronal apoptosis in aged rats

**Figure 5.**
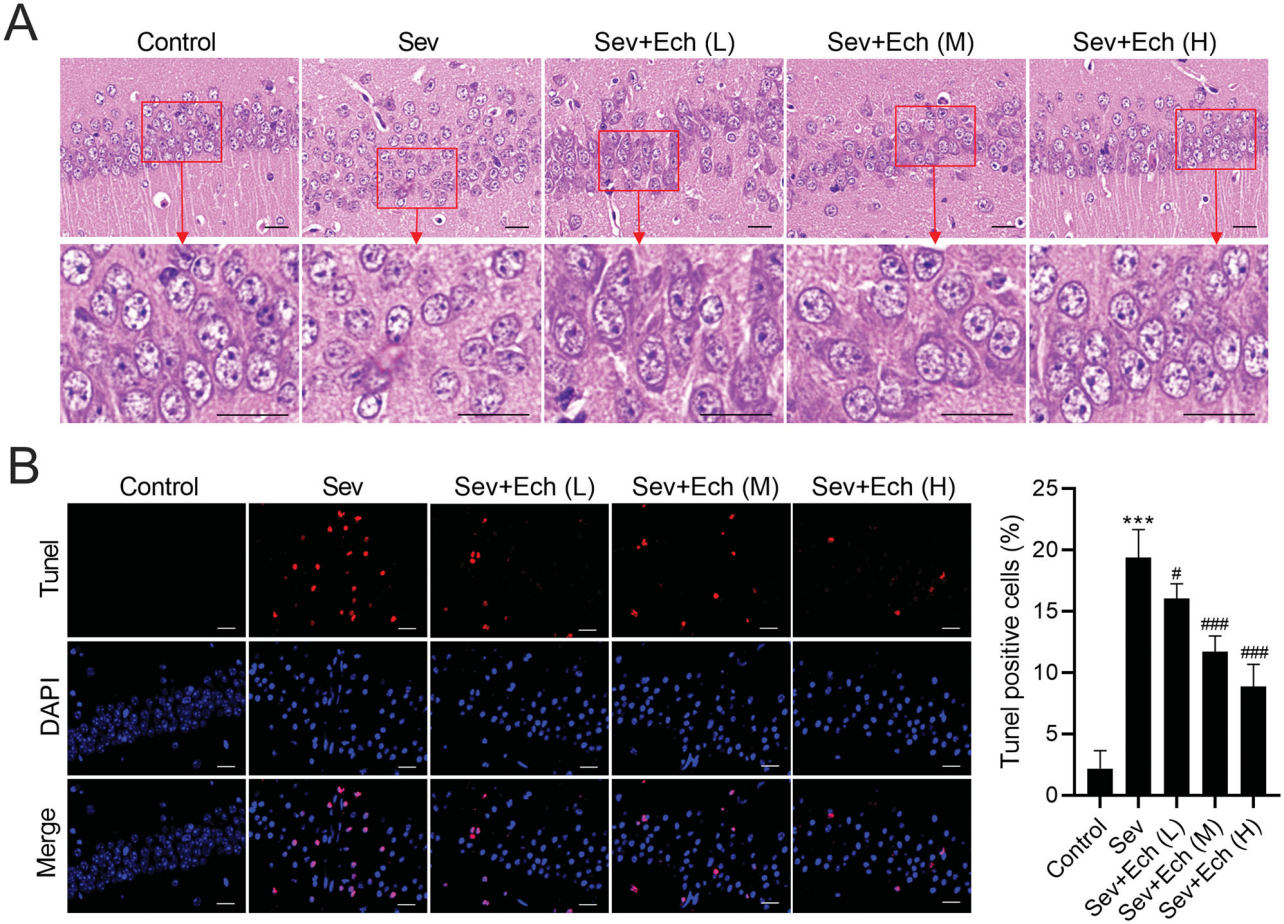
Ech mitigates Sev-induced hippocampal neuronal apoptosis in rats. (A) The cell morphology of neurons in hippocampal CA1 region of rats was assessed by HE Staining. (B) The apoptosis of neurons in hippocampal CA1 region was assessed by Tunel assay. Compared with the control group, ***p < 0.001. Compared with the Sev group, ^#^*p* < 0.05, ^###^*p* < 0.001.

Then, we investigated the effect of Ech on hippocampal neuronal apoptosis in rats. We found that the hippocampal neurons of rats in the control group had clear contours and compact structures. After Sev treatment, the cell morphology had a series of abnormal changes, such as blurred contours, loose structures and so on. However, the damage of hippocampal neurons induced by Sev treatment was attenuated by Ech treatment (Figure 5(A)). In the *in vivo* experiments, Sev treatment caused the hippocampal neuronal apoptosis in rats, whereas this effect was attenuated by Ech treatment (Figure 5(B)). These data demonstrated that Ech alleviated hippocampal neuronal apoptosis in rats with Sev treatment.

### Ech mitigates Sev-induced oxidative stress and ferroptosis in hippocampus of aged rats

**Figure 6.**
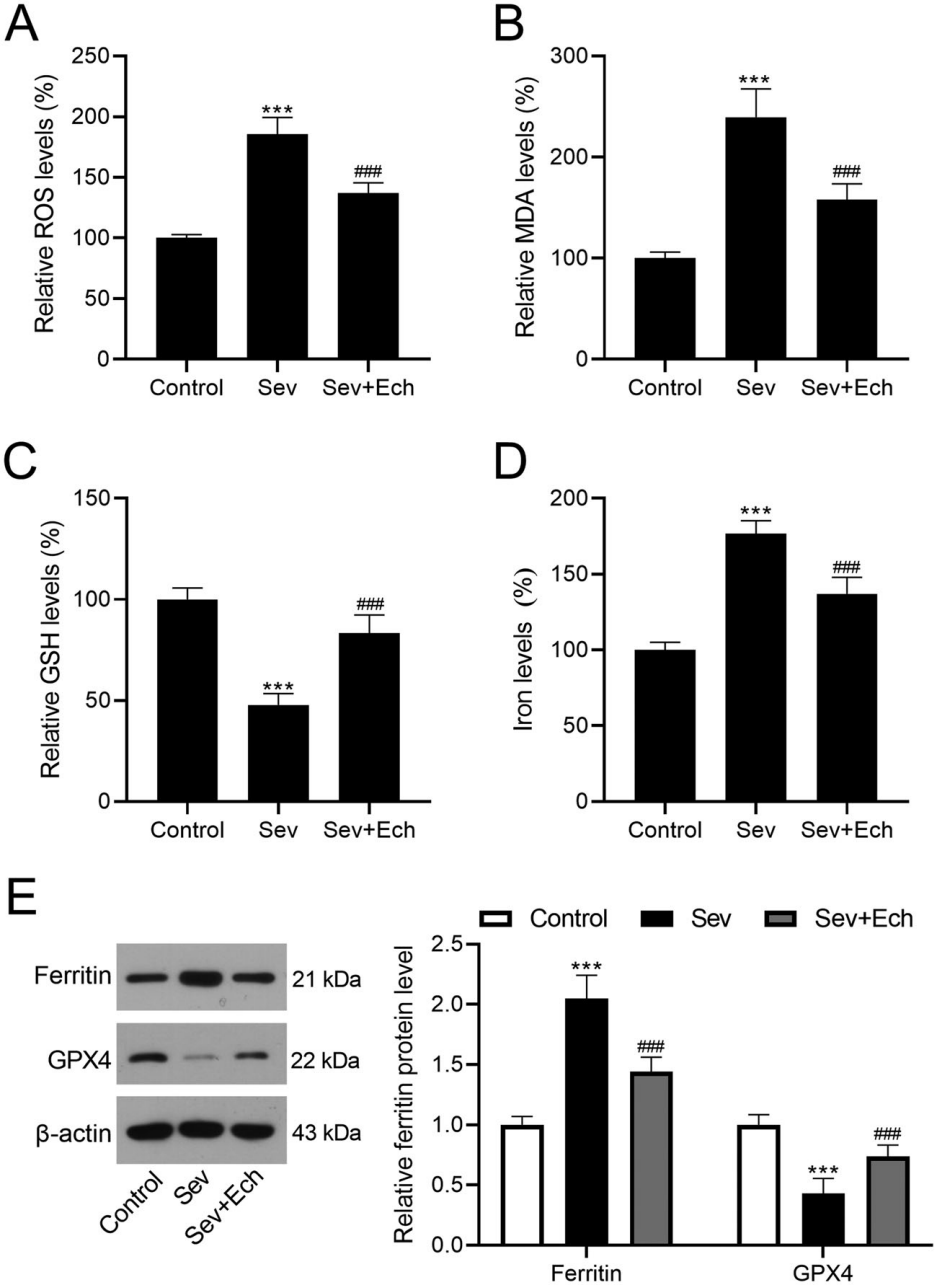
Ech mitigates Sev-induced oxidative stress and ferroptosis in hippocampus of rats. (A) The ROS level was evaluated by a commercial kit. (B-D) The MDA, GSH, and iron levels were detected by commercial kits. (E) The ferritin and GPX4 protein levels were detected by western blot. Compared with the control group, ***p < 0.001. Compared with the Sev group, ^###^*p* < 0.001.

We further investigated the influence of Ech on cell oxidative stress and ferroptosis in hippocampus of rats with Sev treatment. In the *in vivo* experiments of this part, the injection single dose of Ech was 80 mg/kg. We found that the levels of ROS (Figure 6(A)), MDA (Figure 6(B)), and iron (Figure 6(D)) in hippocampus tissues of rats were elevated by Sev treatment, while decreased by Ech treatment. However, the GSH level in hippocampus tissues of rats was decreased by Sev treatment, whereas increased by Ech treatment (Figure 6(C)). Moreover, the increase in ferritin level and the decrease in GPX4 level were observed in hippocampus tissues of rats with Sev treatment, while these changes were attenuated by Ech treatment (Figure 6(E)). Therefore, we speculated that Ech mitigated Sev-induced oxidative stress and ferroptosis in hippocampus of rats.

### Ech mitigates Sev-induced cognitive deficits in aged rats

**Figure 7.**
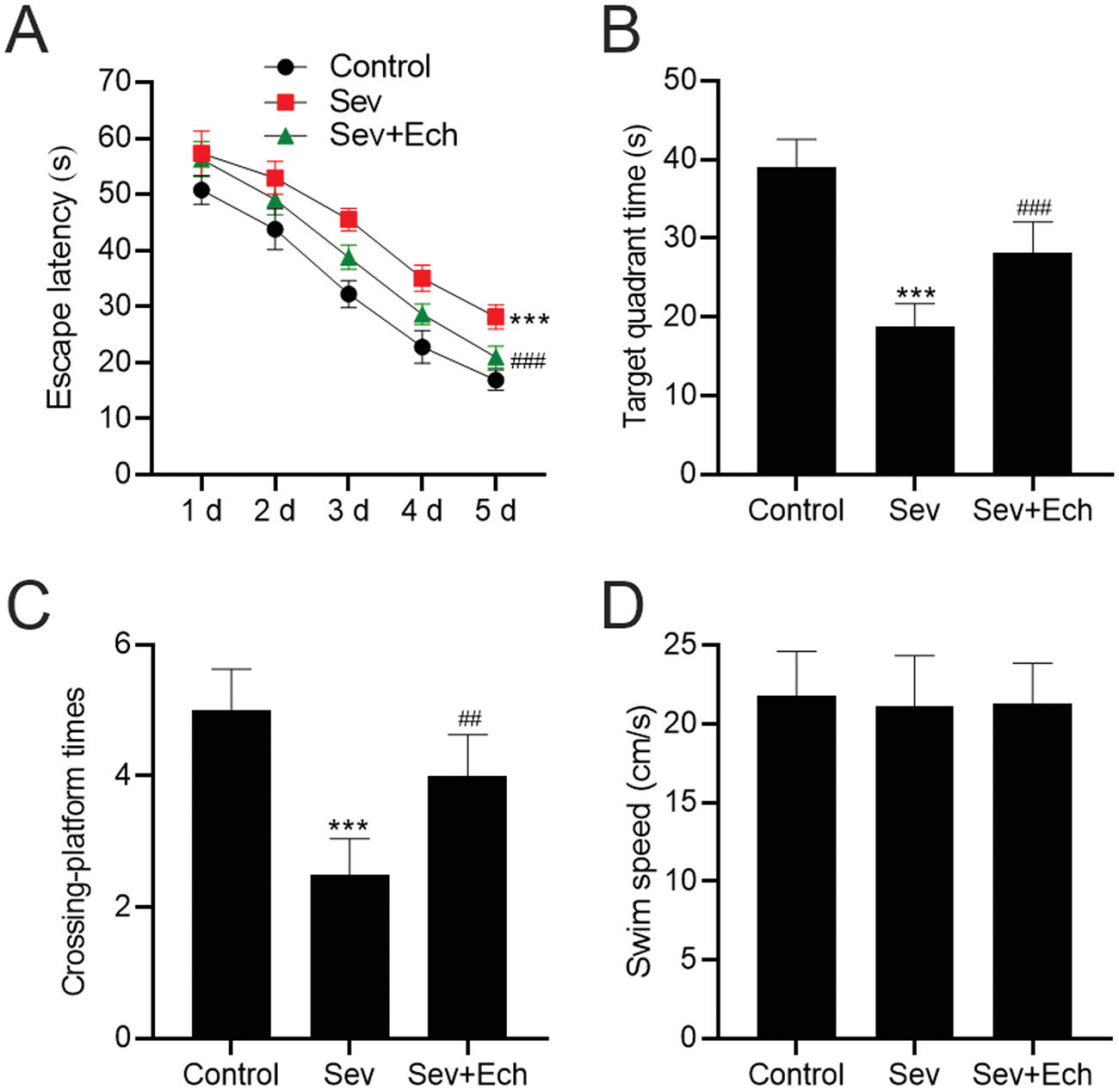
Ech mitigates Sev-induced cognitive deficits in rats. (A) The rat escape latency, (B) time spent in the target quadrant, (C) crossing platform times within 60 s, and (D) swim speed were assessed by MWM assay. Compared with the control group, *p < 0.05, ***p < 0.001. Compared with the Sev group, ^#^*p* < 0.05, ^##^*p* < 0.01, ^###^*p* < 0.001.

To confirm the effect of Ech on Sev-induced cognitive capacity in rats, we carried out the MWM assay. The escape latency was increased after Sev anaesthesia, but this change was attenuated by Ech treatment (Figure 7(A)). Accordingly, the time spent in the target quadrant (Figure 7(B)) and crossing platform times within 60 s (Figure 7(C)) were both decreased in rats anaesthetised Sev, while this influence was attenuated by Ech treatment. Meanwhile, there was no difference in swim speed between the groups (Figure 7(D)). These results indicated that Ech could inhibit Sev-induced cognitive deficits in aged rats.

## Discussion

Sev is widely used in clinical practice because of the early awakening time of patients anaesthetised with it. In addition, Sev also has the advantages of aroma and low irritation to the patient’s trachea (Zhang et al. 2016). Nevertheless, Guo and colleagues revealed that repetitive expose to Sev impaired the learning and memory abilities of old male rats, accompanied by biochemical changes in the hippocampus (Guo et al. 2018). What is more, Tang and colleagues demonstrated that frequent exposure to Sev-based anaesthetic caused neuropathological changes in the brain and long-term cognitive deficits in humans and animals (Tang et al. 2020). Therefore, it is essential to explore the efficient strategies for keeping neurons from Sev-induced nerve injury. We found that Ech attenuate Sev-induced neurotoxicity and cognitive deficits by mitigating oxidative stress and ferroptosis. It is significant to validate the potential regulatory mechanism by which Ech protecting neurons against Sev-induced damage.

The hippocampus is a key part of the brain for learning and cognition (Lisman et al. 2017). In this study, we chose primary rat hippocampal neurons and aged rat model to research the effect of Ech on Sev-induced neurotoxicity and cognitive deficits. Firstly, we tested the toxic influence of Sev on hippocampal neurons *in vitro* and *in vivo*. Sev treatment decreased cell viability but contributed to apoptosis, oxidative stress, and ferroptosis in hippocampal neurons and hippocampus of aged rats. These results were in line with preceding studies (Zhang et al. 2020; Apai et al. 2021). Besides, Ech has been proved to reduce LDH level and attenuate apoptosis in the cardiomyocyte of myocardial ischemic/reperfusion mice (Niu et al. 2020). In addition, Liang and colleagues demonstrated that Ech played an antioxidant role (Liang et al. 2018). But so far, the relationship between Ech and cell ferroptosis is still unknown. Herein, we found that Ech increased cell viability but decreased apoptosis, oxidative stress, and ferroptosis in hippocampal neurons and hippocampus of rats anaesthetised with Sev. These results were similar to previous studies and for the first time demonstrated the inhibitory effect of Ech on cell ferroptosis (Liang et al. 2018; Niu et al. 2020). Subsequently, we demonstrated the regulatory effect of Ech on the Nrf2 signalling.

Nrf2 is a very important transcription factor and involved in regulating cellular defense against toxicity and oxidative damage (He et al. 2020). In addition, it has been illustrated that the activation of Nrf2-mediated antioxidant signalling could protect neurons from oxidative injury (Wei et al. 2020). Additionally, Ran et al. (2021) found that Ech could modulate Nrf2 signalling in HLECs to exert antioxidant function. In this study, Ech upregulated Nrf2 expression in *in vitro* and *in vivo* models of anaesthesia nerve injury. Furthermore, Ech mitigated neurotoxicity in hippocampal neurons with Sev treatment by modulating Nrf2 signalling, which was alike with the results of a previous study (Ran et al. 2021). Further, we demonstrated that Ech attenuated Sev-induced cognitive deficits in rats. Effects of Ech on Sev-induced behavioural and memory injury *in vivo* should be explored in future. At the same time, this study also has some shortcomings. We only used a cell model and a rat model for analysis and study, and clinical practice was absent. In the future research, we will carry out further verification in clinical practice.

## Conclusions

According to this research, Ech could mitigate Sev-induced apoptosis, oxidative stress, and ferroptosis in hippocampal neurons and hippocampus of rats by activating Nrf2 signalling. Moreover, Ech improved Sev-induced cognitive deficits in aged rats. These findings suggested that Ech may be developed as a neuroprotective agent to reduce Sev-induced neurotoxicity in the clinic. However, the relationship between Ech and Sev still needs further investigation and studies.

## Author contributions

Fujun Li conceived this study. Zilong Xu, Yanqiu You, Qiuqin Tang and Hui Zeng performed experiments. Tianshou Zhao and Juan Wang analysed the data. Zilong Xu wrote the manuscript. Fujun Li reviewed and modified the manuscript. All the authors read and approved the final manuscript.

## Data Availability

All data generated or analysed during this study are available from the corresponding author upon reasonable request.
